# Crick A Mind in Motion by Matthew Cobb, New York, Basic Books, 2025, 595 pp., $36.00 U.S. (Hardback), ISBN 9781541602885.

**DOI:** 10.1080/19491034.2026.2642527

**Published:** 2026-04-07

**Authors:** Thoru Pederson

**Affiliations:** Department of Biochemistry and Molecular, Biotechnology, University of Massachusetts Chan Medical School, Worcester, MA, USA

Having reviewed a previous biography of Francis Crick on these pages [1], the editors generously offered me the opportunity to do so once again. I had been aware that Matthew Cobb was writing this and although we e-mailed one another a few times, I do not feel any conflict in reviewing what he has produced.

**Figure:**
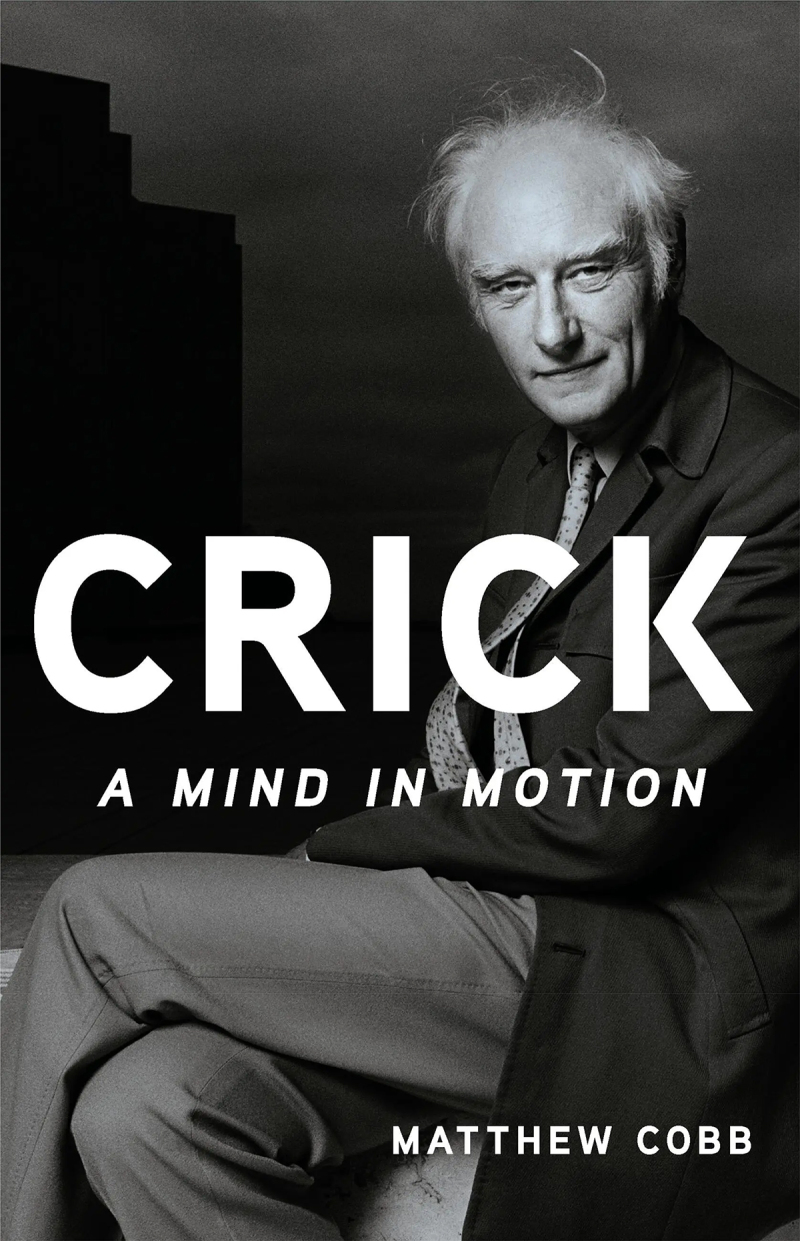

There have been two previous biographies, a short one by Matthew Ridley [2], sort of in the vein of what in Britain is often called a ‘primer,’ and the other by Robert Olby, in full form [3]. Although Cobb’s should of course be assessed on its merit, the earlier two works make for an unavoidable comparative lens.

Cobb took a proper historian of science tack, for he is one, and dug deeply and productively into the two major collections of Crick’s papers, as well as many other sources. The result is an encyclopedic array of vignettes, many not previously published. The writing style is fluid- no passage suffers from lexical desiccation nor from syrupy excess.

The biographical details of Crick’s family, early schooling and war service (studying mines) are done very well. Cobb is not shy about emphasizing Crick’s romantic dalliances while married and, on the other hand, describes his second wife, Odile (nee Speed) Crick, in engaging depth for the impressive artist and intellectual that she was. I will return to Cobb’s coverage of Crick’s behavior toward women as well as hisaffinity for eugenics.

**Figure 1.**
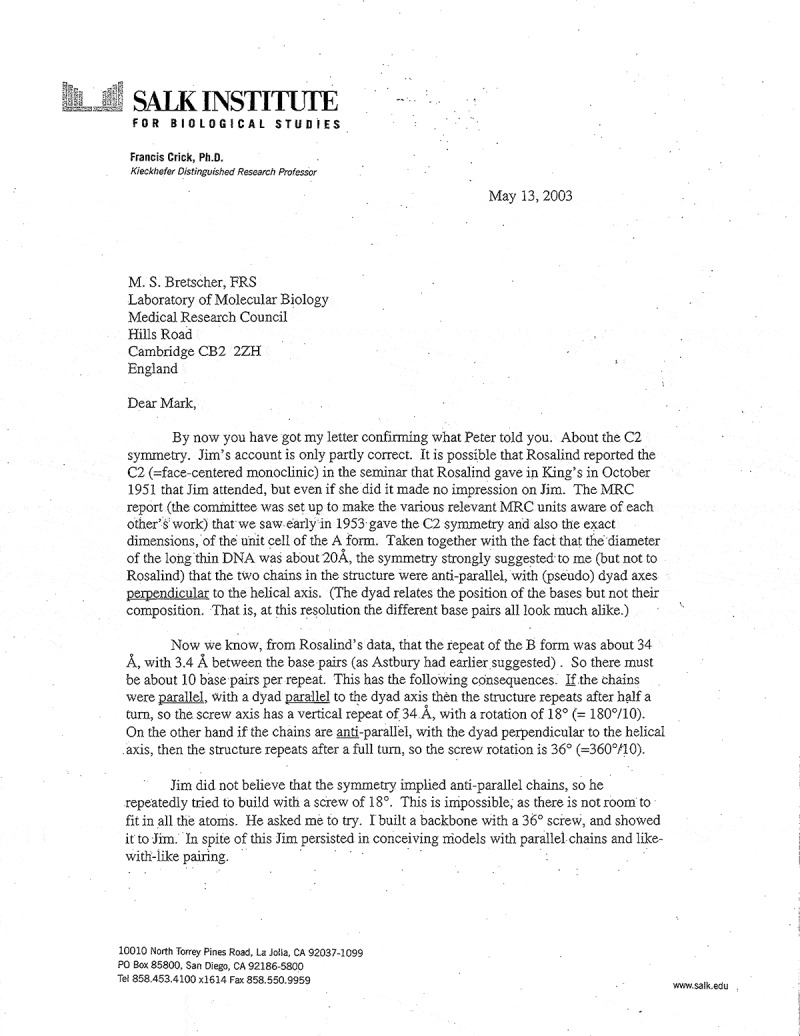
The letter that Crick wrote to Mark Bretscher. GBR/0014/BRTS 42/73, Mark Bretscher Archive, Churchill archives Centre, Churchill college, Cambridge. Item 73: https://archivesearch.lib.cam.ac.uk/repositories/9/archival_objects/422577.

As to the double helix, a key virtue of the book is that Cobb found a particular letter (not present in either of the known collections) that Crick wrote late in his life (2003) to a younger Cambridge colleague (Figure 1). The letter emphasizes Crick’s view on the degree to which Watson was obdurate to the available information and how, instead, Crick seized upon it and built models to see two alternative geometries of the DNA helical structure. However, in recent discussions, I’ve had with Matthew Meselson (Harvard University) and Alex Gann (Cold Spring Harbor Laboratory), an alternative view arose, viz. that, Watson, in fact, was very astute about the structural information at hand and had been keenly building models all along. This is nowhere more evident than in the words of Watson himself [4]. Thus, we might view the letter as Crick downplaying Watson’s role at this stage.

It is widely conceded that a major clue in Crick’s and especially Watson’s early efforts were acid-base titrations of DNA done in 1947 by John Gulland and colleagues [5], which made the existence of hydrogen bonds among the bases very probable. I was surprised that Cobb didn’t mention this important point, at least *en passant.*

Ridley, Olby and Cobb alike had the task of describing Crick’s post-DNA career. The first thing Crick had to do after the double helix was to finish his PhD project. It was in the thesis work that he had completed earlier that he had seen a hemoglobin fold-back region that, later, alerted him to the likely meaning of Rosalind Franklin’s data on the DNA unit cell.

Cobb’s description of how Crick and Sydney Brenner then demonstrated that the genetic code is based on triplets is comprehensive and accurate, and he goes on to describe Crick’s next research on pattern formation in insect epidermis, motivated by Lewis Wolpert and in collaboration with Peter Lawrence. This did not lead to anything other than theoretical models for signal molecule gradients.

As many readers of *Nucleus* know, Crick then became enthusiastic about the structure of chromatin. Here Cobb goes into more detail than other biographers, including an account of Crick’s prescient ideas on the roles of repetitive DNA in gene regulation, and then his embarrassing model of chromatin structure, soon supplanted by what proved to be the correct one by Roger Kornberg and Jean Thomas. (I could have wished Cobb had mentioned the latter.)

As to the subsequent career, Cobb, similar to the other biographers, describes how Crick and his wife moved to the Salk Institute but provides more detail. Some friends and colleagues had assumed that they made a precipitous decision to leave England for sunny southern California, this in part because of Jonas Salk’s well-publicized plan to have his new institute include resident ‘fellows,’ who would walk about the grounds – perhaps not wearing don’s robes of classical days, but just being there to inspire the thinking of the institute’s scientists. In her book about the founding and early years of the Institute [6], Suzanne Bourgeois describes this quixotic vision of Salk with empathy. But Crick never became a permanent fellow, despite entreaties from Salk and the trustees. But in due course, 1977, a professorship funded by the Kieckhefer Foundation was offered to Crick and he accepted. It is a credit to Cobb’s research that he has provided this accurate account of the Cricks’ ‘trip west.’

In his treatment of the work on consciousness Cobb describes how Crick got to a realization that this emergent property of the mind was, at least at the time, beyond reach but that perhaps vision was accessible. Cobb provides the best description of Crick’s challenges on this front that I have seen. At a meeting in 2018 at Trinity College, Dublin celebrating the 75^th^ anniversary of Erwin Schrödinger’s famous lectures there, I had the pleasure of meeting Cristoph Koch, Crick’s important collaborator in his consciousness work. He told me how much Crick had meant to him and that he viewed their work as a step, not a failure. Although Crick of course describes his work on consciousness engagingly in his autobiography [7], Cobb has done a superb job of expanding on its various tributaries and the degree of penetration of the problem. Cobb also reveals something from Crick’s La Jolla days I had not known, about the ‘Central Dogma.’ This term always seemed right to me and most others, especially Brenner’s famous quip that ‘once the information gets into protein it cannot get out again.’ But Cobb reveals that Crick intensely disliked it in the form being used by Watson and others. Cobb describes forays Crick had on this both with Watson and John Maddox, the editor of *Nature*, and emphasizes Crick’s assertion that the Central Dogma was actually a negative statement, akin to Brenner, *viz*. that information in a protein cannot proceed to DNA or RNA. I found this section quite engaging and do not think other biographers have dealt with it as well.

Both Cobb and Olby conveyed one of Crick’s particular traits, namely how he was usually rather stand-offish until he found reason to think a person ‘was OK.’ I experienced this personally during his visit to the Worcester Foundation in 1972, when we had not previously met. In my office, I told him about my experiments on how chromatin structure changes during G1, S and G2 in cycling cells. I mentioned that *Nature* had returned my paper with a partial snippet of the (sole) reviewer’s comments scotch-taped onto the letter. Crick quickly angered and said: ‘I told them to accept your paper!’ By this time, I had published it elsewhere [8], but I will always remember how irritated he was. Not that I had been his trainee or had ever even been at Cambridge, but his reaction just out of what I sensed was a constitutional kindness when his assessment of the person and facts warranted.

For me, a very important aspect of the biography, and a credit to Cobb, is the descriptions of Crick’s well-known libertine attitudes, inappropriate treatment of women (in one incident, an act of sexual harassment of an undergraduate student when he was visiting Jim Watson’s lab) and his often-troubling views on eugenics. These failings had not been adequately addressed by other biographers and must always be part of the record.

Three biographies of this great scientist are now on my shelf, along with Crick’s autobiography, which is a very rich source on its own (some autobiographies not being so). To say which biography of Crick is the most valuable depends. For a most pleasurable brief account, there is nothing better than Ridley. His strikes me as exactly how James Gleick captured Newton in the ‘primer’ mode [9,10], leaving Westfall’s previous account in iconic status [11]. For those who want to know both the science and the man, there is Olby’s fine biography. But I believe Cobb’s superb effort now holds the highest ground, and I doubt it will soon or ever be displaced.
